# Supplementary material for: Are carbon emissions trading and green financial instruments synergistic? -Comprehensive quantitative research based on content analysis
Source: PLoS One. 2024 Mar 7;19(3):e0298601. doi: 10.1371/journal.pone.0298601 (PMC10919601; doi:10.1371/journal.pone.0298601)
Supplement: S1 Data — (ZIP) [file pone.0298601.s001.zip › Data/地级市Z_F工作报告.zip!/╕╩╦α/╛╞╚¬╩╨/└┤╫╘╣1⁄2╓┌║┼í╛┬φ┐╦╩2╛▌═°í┐_2010.docx]

各位代表：

　　现在，我代表酒泉市人民政府向大会作工作报告，请予审议，并请各位政协委员和列席人员提出意见。

　　2009年工作回顾

　　2009年，是新世纪以来我国经济社会发展最为困难的一年，面对国际金融危机的冲击和严峻复杂的经济形势，市政府在中共酒泉市委的正确领导下，坚持以科学发展观为指导，认真贯彻落实党的十七届三中、四中全会精神和省委、省政府的工作部署，坚持重点抓风电，难点抓移民，整体推进经济社会各项事业全面发展，较好地完成了市二届人大五次会议确定的任务目标，实现了跨越式发展"五大突破"。风电装机突破200万千瓦，达到220万千瓦；风电装备制造业销售收入突破50亿元，达到64亿元，占全省装备制造业销售收入的80%；移民工作取得突破性进展，争取扶贫开发资金1.3亿元，是2008年的11倍；生产总值突破300亿元，达到321亿元，增长14.7%；财政总收入突破45亿元，增长1.07倍，实现当年翻番，生产总值和财政收入增速居全省第一，总量跃居全省第二。完成全社会固定资产投资300.16亿元，增长91％；社会消费品零售总额75.83亿元，增长21.3%；城镇居民人均可支配收入13704元，农牧民人均纯收入6410元，分别增长10.2%和11.2%；政府承诺的创业促就业、安全饮水、危房改造等10件惠民实事得到较好落实。一年来的主要工作及成效是：

　　1、抓项目，经济增长明显加快。认真落实国家扩大投资、启动内需、振兴产业、促进经济平稳较快发展的一揽子计划，积极争取国家投资项目，加快开发优势产业项目，扶持民间创业项目，强化领导包抓、部门包挂、协作攻关、绩效考核工作机制，项目建设取得明显成效。全年开工建设项目1153项，增加219项，其中投资亿元以上项目68项，5000万元以上项目121项，千万元以上项目449项。中央新增4万亿元投资计划下达后，全市各级按照"快选、多选、选好"项目的要求，争取项目200项、投资9.01亿元，项目开工率达到86%。重大基础建设项目取得新进展，敦煌水资源合理利用和生态保护项目通过水利部审查，玉门市列入全国第二批资源枯竭转型城市，敦格铁路获国家批准立项，嘉红铁路电气化改造正式启动，酒航铁路完成环评、水保、土地预审等前期工作，酒火公路、敦七公路改造工程完工，瓜猩高速、敦当公路开工建设，新建、改建农村公路1407公里。招商引资成效显著，组团参加了兰洽会、西洽会等重点节会，签约开工招商引资项目127个，建成投产103个，引进到位资金87亿元，增长1.2倍。全市固定资产投资增速同比提高55.5个百分点，有力地拉动了经济增长，实现了保增长的预期目标。落实积极的财政政策和适度宽松的货币政策，加快财政支出进度，完成财政总支出49.56亿元，增长28.1%；扩大信贷投放规模，年末金融机构贷款余额148.87亿元，增长40.3%；证券市场完成交易额175.71亿元，增长59%；大禹节水公司在创业板成功上市，融资2.52亿元。

　　2、攻风电，工业生产企稳回升。面对外部市场急剧萎缩、工业生产低位徘徊的态势，坚持以风电开发为牵引，促调峰电源、光电、网架和装备制造业发展，实施"一轴两片五大主导产业"发展战略，加大传统产业技改力度，工业生产和经济效益逐步回升，全市完成工业增加值122.8亿元，增长13.8%，实现利税21.44亿元，增长2.04倍。千万千瓦级风电基地建设全面启动，一期380万千瓦风电项目全面开工，二期755万千瓦风电完成预可研，引进18家上市公司参与风电场建设，全市风电装机规模达到220万千瓦，新增169万千瓦。750千伏输变电工程和酒泉热电厂开工建设，敦煌、柳园330千伏变电站完成选址。光电产业规划通过评审，国投华靖敦煌光伏电站投产发电，金塔13兆瓦光伏发电项目批准立项。装备制造业迅速崛起，22家国内风光电装备制造企业落户酒泉，其中上市公司14家，开工建设项目12个，中复连众风机叶片和金风科技、华锐科技风机总装等5个项目建成投产。矿产开发秩序进一步规范，煤炭、黄金矿点由30个整合为12个，矿产资源开发秩序整顿工作受到国家表彰奖励。配套完善工业园区基础设施，组建园区绿化公司和环卫保洁公司，启动行政服务大厅，西园集中供热一期工程、四湖一带景观工程建成完工，园区综合服务功能进一步增强。

　　3、调结构，特色农业增产增效。把调优、调强特色产业作为推进"一特四化"的重要举措，编制实施六大特色产业发展规划，全市粮食面积增加6.1%，棉花面积减少24.2%，新增设施蔬菜、网室制种、特色林果等高效产业面积22.4万亩，总面积达到122万亩，其中建成万元田8.8万亩、五千元以上高效田18万亩，结构调整的幅度及产生的效益是近年来最明显的一年。积极推进牛羊产业大县建设，提高规模化、集约化、标准化养殖水平，调引良种羊5.5万只，新建、改建标准化养殖小区73个，累计达到816个,规模养殖量占饲养总量的62.6%，全市新增肉牛1.1万头、肉羊58万只，牛羊饲养量分别达到18.4万头和505万只。加强动植物疫病防控，近两年未发生重大动物疫情。全面推广标准化生产技术，完成无公害农产品产地认定、产品认证15个，农产品质量安全合格率达到98.5%。扶持发展农产品加工龙头企业，新建、扩建农产品加工项目58个，全市规模以上龙头企业占全省1/5，辐射带动生产基地148.9万亩、农户13.5万户。注册登记农民专业合作社237个，增长2.65倍，19个专业合作社进入全省百强行列。组织开展劳务技能培训5.67万人，输转农村劳动力13.53万人，劳务收入占农民人均纯收入的1/4。积极推进农村土地流转，新增流转面积5.5万亩，累计达到17.4万亩。新农村建设深入推进，编制完成12个乡镇、100个行政村建设规划，整体改造示范点160个，建成小康住宅示范点64个，硬化人行道106公里，新建沼气池1.84万座，阿克塞县被评为全国首批农村社区建设示范单位。支农力度进一步加大，财政用于"三农"支出11.52亿元，增长1.17倍,共发放惠农补贴资金1.8亿元，农民人均受益416元。

　　4、创名城，城市品位不断提升。巩固旅游、双拥城市创建成果，深入开展环保、卫生、园林、文明城市创建活动，按照"高起点规划城市、精细化管理城市"的要求，增强城市的聚集、辐射功能。成立城市规划委员会，编制酒泉市城市总体规划和市域城镇体系规划，瓜州、敦煌、肃北、阿克塞启动修编新一轮总体规划，全市建制镇和75%的乡完成规划编制，村庄规划覆盖率达到51.2%。完善市政基础设施，重点实施了酒泉市区金泉路、阳关路建设工程和肃州南路、北环东路改造工程，酒泉医疗废物处置工程基本完工，污水处理改造升级项目完成土建工程。编制城市绿地系统规划，完成新城区梧桐园、世纪大道北段和安康路南段绿化工程。理顺城市管理体制，下划城市执法监察和物业管理监督职能，发挥社区在城市管理中的作用，开展市容市貌整治活动，城市管理水平进一步提高。县市城区集中供热、园林绿化、道路建设步伐加快，共投资14.6亿元，完成供热分户计量和节能改造1.14万平方米，新增公共绿地175万平方米，绿化覆盖率达到34%，人均公共绿地10.3平方米。玉门和金塔垃圾处理、敦煌和肃北城市广场、瓜州老城区改造等重点工程加紧实施，阿克塞创建为全国文明县城。

　　5、促消费，商贸旅游日趋活跃。充分发挥消费对经济增长的拉动作用，加快区域性商贸中心建设。制定城市商业网点规划，加快时代广场、春光市场改造和瓜州新州里、玉门综合市场建设，吸引知名品牌店、直销店、加盟店入驻酒泉，用品牌效应提升消费信心。开发农村市场，启动"家电下乡"销售网点173个，销售家电2.3万台、汽车和摩托车2493辆，兑付补贴1081万元；继续实施"万村千乡"市场工程和"便民服务"工程，新建改造农产品市场和配送中心17个、农家店251个，农家店累计达到1028个，实现乡村全覆盖。积极发展会展经济，举办美食、洋葱、酒花等特色节会，引办、承办国内外会议90场次，参会人数1.7万人，带动消费5000多万元。全市商品零售额达到75.83亿元，人均7443元，是全省人均水平的2倍。积极开拓国际市场，扩大进出口规模，实现进出口总额6424万美元，增长23.4%。加快旅游资源开发和配套设施建设，实施旅游项目44个，完成投资4.06亿元。敦煌风情城、玉门铁人纪念馆、瓜州桥湾城、阿克塞金山湖景区、肃北冰川游客接待中心建成，新增国家3A级旅游景区3处，星级饭店10家，绿色饭店18家，建成星级厕所11座，敦煌市被评为国家精品旅游城市。积极拓展旅游市场，刺激旅游消费，举办"敦煌艺术展"，组团参加重大旅游推介会，编辑发行酒泉旅游手册，组织开展"酒泉人游酒泉"活动，休闲度假和乡村旅游不断升温。全市接待海内外游客348.5万人次，增长14.3%，实现旅游收入23.6亿元，增长27.6%，占GDP比重达到8%，提高0.3个百分点。

　　6、护绿洲，生态建设扎实推进。大力推进农业高效节水工程建设，新增节水灌溉面积1.97万亩，累计达到12.4万亩。敦煌万亩节水示范园区列入全省节水示范点。强化用水计量，实施机井智能化控制，新安装智能水表4670块，累计达到8189块，安装率达到80%，累计关井412眼，压田4.46万亩，地下水开采量逐步减少，水资源向高效、节水、生态领域配置。全面启动集体林权制度改革，广泛开展造林绿化活动，完成人工造林15.7万亩，绿色通道438公里，防风固沙5.3万亩。加强环境监测，重点污染源自动监控中心建成使用，我国首个戈壁荒漠生态与环境研究站落户敦煌。实施集中供热、电机节能、余热利用等重点节能项目28项，万元GDP能耗1.514吨标准煤，下降5.4%。推广应用新型墙体材料，控制使用粘土实心砖。落实减排措施，对59户重点企业进行目标监控，玉门炼化厂、祁连山制药厂和同福化工厂污水处理技改工程建成，全市减排二氧化硫150吨、化学需氧量1200吨。加强城市空气、环境水质治理，在85户餐饮企业实施污水前端防控，在全省抽样调查中，酒泉公众城市环保满意率达到84.5%，提高8个百分点。耕地和基本农田得到有效保护，实现耕地占补平衡。

　　7、增投入，社会事业协调发展。加大社会事业投入，全市用于科教文卫体等方面的支出13.4亿元，增长30.2%。按照"教育资源相对集中、医疗资源切实下沉"的思路，推进公共服务均等化。进一步改善办学条件，酒泉市第四、第五中学完成教学楼主体工程，酒泉卫校教学楼、酒泉特殊教育学校及玉门寄宿制初中、瓜州三中、金塔四中基本建成，全市新建寄宿制学校公共浴室46座。推动新一轮农村学校布局调整，18个乡镇中小学完成布局调整任务，全市农村高中生和25个乡镇初中生实现进城就读。狠抓教育信息化建设，建成信息技术教育示范学校20所，90%的学校连接教育宽带网。二类城市语言文字评估达标通过省政府验收，我市被评为全国推进义务教育均衡发展先进地区，酒泉职业技术学院列入全国示范性高职院校建设计划。完善公共卫生服务体系，基本实现初级卫生保健目标。酒泉市医院门诊大楼、市二院住院部大楼及基层卫生院所等59个重点项目开工建设，金塔县医院住院部大楼建成使用。扶持发展中医药事业，设立中医药发展专项基金，中医床位补贴提高50%。实行乡村医疗服务一体化管理和乡镇卫生院药品"零差价"试点。有效防控甲型H1N1流感传播，严密防范鼠疫疫情。认真落实计划生育利益导向三项制度，创新工作机制，我市被国家确定为人口与计划生育综合改革示范市。制定完善加快科技进步的意见，组织实施科技计划项目167项，申请专利135件，80项科技成果通过鉴定，科技对经济增长的贡献率达到49%。文化事业繁荣活跃，《酒泉通史》完成初稿，酒泉图书馆、飞天数字影院和丝绸之路博物馆建成使用，建成乡镇综合文化站10个，新建农家书屋220家，村级覆盖率达到90%以上。五个市县区博物馆免费开放，每年补贴资金850万元，敦煌莫高窟、金塔大湾城等文物保护项目开工建设，第三次文物普查完成野外勘查，新发现文物点631处。扩大数字电视覆盖范围，数字电视信号传输驻酒空军部队，完成广播电视"村村通"年度任务，纪录片《敦煌书法》在央视播出。广泛开展群众性体育活动，全民健身活动中心完成规划设计，举办全国青少年轮滑赛，组织参加全省残疾人田径锦标赛并取得优异成绩，酒泉体校跻身全国体校200强。着力维护社会稳定，深入开展安全生产标准化建设，165户企业达到三级安全标准，继续对重点领域进行专项治理，安全生产形势保持稳定。集中开展矛盾纠纷排查化解和"信访积案化解年"活动，畅通信访渠道，群众来信来访、集体上访和越级上访大幅下降。广泛开展依法治理和"法律七进"活动，启动"三调联动"机制，建立法律援助工作站112个，规范化司法所创建率达到53%。加强社会治安综合治理，开展"督察风暴年"活动，治安管理、维护稳定和打击犯罪工作名列全省前列。改善公检法司基础设施条件，开工建设"三基"项目99个，检察院业务技术用房和一批基层派出所、法庭相继建成，完成4个武警中队营房建设任务，消防指挥中心建成。双拥、人防和边防工作深入推进，军地"双十"工程进展顺利，酒泉国防教育基地被全国首批命名，酒泉人口疏散基地和马鬃山边防服务中心开工建设。统计监测服务水平提高，完成第二次经济普查。民族团结进一步巩固，承办全省民族团结进步项目帮扶现场会，阿克塞县被评为全国民族团结进步模范集体，防震救灾工作荣获全国先进，妇女、儿童、台侨、宗教、档案、气象等工作取得新的成绩。

　　8、重民生，惠民实事较好落实。认真实施积极的就业政策，选拔500名纯农（牧）户零就业家庭本科生到农村中小学和乡镇卫生院工作，购买公益性就业岗位1412个，城镇新增就业1.12万人，下岗失业人员再就业5006人，城镇登记失业率控制在3.35%。广泛开展"151"创业促就业行动，全市新增小额贷款担保基金1505万元，为1408名创业人员和20户企业发放贷款7363万元，带动1.2万名劳动者实现就业，全市私营企业达到3996户，个体工商户达到32485户，分别增长12.5%和8.6%，我市被国家列入创建创业型城市试点。构筑城乡居民生活基本保障安全网，全市用于社会保障和就业支出11.65亿元，增长53.1%，城镇职工参加基本养老、基本医疗、失业、工伤保险30.8万人（次），增长10%，年基金收入突破4亿元，企业退休人员平均每人每月增加养老金120元，人均增加采暖费补贴550元。解决了1360名被辞退民办教师生活补贴和579名集体企业职工养老保险问题，村干部养老保险实现全覆盖。调整城乡居民基本医疗政策，提高住院统筹和大病补助标准，城镇居民基本医疗和新农合参保率分别达到94.8%和95.1%。金塔、阿克塞两县列入全国首批新型农村社会养老保险试点县，全市2035名破产企业退休人员纳入医疗保险，玉门老市区职工群众就业、医疗、社保等遗留问题得到妥善解决。完成城乡低保和农村五保供养提标扩面工作，发放保障金1.7亿元，保障人数16.8万人。拨付救助资金，解决特困群体涉案97件。深入开展扶贫济困活动，大力发展慈善、福利事业，5个农业县市全部建立了慈善机构，募捐资金230万元，救济灾民和特困户7.52万户，福利彩票年销量突破亿元，一批农村敬老院、残疾人服务中心、儿童福利院开工或建成。编制完成农村饮水安全工程五年建设规划，又解决了12.5万名农村居民安全饮水问题，其中疏勒河项目移民5.51万人。改造农村危旧住房13546户、农村贫困户和残疾人住房2809户。新增廉租房3933套、经济适用住房1012套，发放租赁补贴10620户，人均住房10平方米以下低收入家庭全部纳入保障范围。

　　9、聚合力，扶贫开发取得实效。按照"规划先行、项目支撑、找准路子、强化措施"的要求，完善领导包挂、部门帮扶责任制，加快移民扶贫开发，市县两级财政列支移民扶贫资金1000万元，争取移民帮扶资金1.3亿元，移民人均纯收入达到1522元，增长15%。编制完成11个移民乡基础设施和经济社会发展十年规划，沙河、梁湖等4个移民乡完成总体规划编制，瓜州县及全市11个移民乡全部列入省级扶贫计划，将8.48万名绝对贫困移民全部纳入低保范围，占移民总数的54.8%。积极争取移民低保、医疗、临时救助及社会捐助"一特四倾斜"扶持政策，改造移民住房1292户，为移民群众发放过冬衣物18.35万件，为11个移民乡镇卫生院配备急救车和医疗设备，选派70名教师和58名医务人员到移民乡开展对口援助。在玉门独山子乡召开帮扶现场会，落实帮扶资金和实物价值1772万元。经过积极争取，省委、省政府已决定今后不再给酒泉安置新的移民。

　　10、强基础，政府效能得到提高。深入开展学习实践科学发展观活动，大力推进政府自身建设。开设"行风面对面"电视专栏，全市15个行政执法部门和窗口服务单位公开接受群众评议，受理政风行风热线投诉475件，办结率100%。深入推进政务公开和公用事业单位办事公开，坚持依法行政和民主决策，制定出台规范性文件22件，对城市规划、拆迁改造、民生工程等重大事项进行评审论证。规范国库集中支付管理制度，136个市级管理预算单位实行国库集中支付，120个行政、事业单位纳入结算中心集中核算。启动新一轮政府机构改革，全面实施事业单位岗位设置管理工作，推行事业单位公开招聘制度和乡镇机构编制实名制管理。快捷办理审批事项，政务大厅服务效能进一步提高，受理办件40.24万件，办结率达到99%。自觉接受人大依法监督和政协民主监督，办理人大代表建议126件、政协委员提案164件，办结答复率达到100%。加强廉政建设，强化行政监察和审计监督，开展了"小金库"、公款出国、民生资金和行政事业收费等专项治理，审计工作受到国家表彰奖励。规范国土资源出让、工程承包、政府采购等招投标程序，实现国土资源竞价收益8.28亿元，增长1.02倍。改善基层组织办公条件，新建、改建67个村委会办公用房，为435个村委会补助办公经费217.5万元，为社区和村组赠订报纸2837份。

　　过去的一年，我们积极应对金融危机的冲击，取得了经济快速增长、社会和谐稳定、民生明显改善的巨大成就，这是我们践行科学发展观，认真贯彻落实中央和省委、省政府及市委战略部署的结果，是市人大、市政协和社会各界大力支持的结果，是全市人民辛勤努力、团结奋斗的结果。在此，我代表市政府向各位人大代表、政协委员和全市广大干部群众，向各民主党派、工商联、人民团体、离退休老同志、驻酒人民解放军、武警官兵、公安干警和中央、省属驻酒各单位以及所有关心、支持酒泉发展的各界人士，表示衷心的感谢和崇高的敬意！

　　在总结成绩的同时，我们也清醒地看到，政府的工作与科学发展的要求还有一定的差距，资源开发和综合利用水平较低；农民持续增收难度加大，移民扶贫开发任务繁重；中小企业融资困难，创业环境仍不宽松；机关办事效率还不能满足群众的愿望，不能完全适应快速发展的要求。这些问题我们必须高度重视，并在今后的工作中努力加以改进。

　　2010年工作思路和目标

　　今年是实施"十一五"规划的最后一年，做好今年的工作对有效应对金融危机、保持经济平稳较快发展至关重要。当前，国内经济回升的基础尚不稳固，内生动力仍然不足，金融危机对我市的影响依然存在，发展中的不确定因素依然较多。我们要审时度势，沉着应对，超前谋划，扎实工作，巩固和开创"保增长、保民生、保稳定"的良好局面。分析国内外经济形势，我们仍面临着许多难得的发展机遇，国家将继续实施积极的财政政策和适度宽松的货币政策，不断完善应对危机的一揽子计划，制定了推进新一轮西部大开发的思路对策，即将出台支持甘肃经济社会发展的政策意见，这些将为我市加快发展创造良好的外部环境；国家把新能源建设作为调整经济结构、转变发展方式的重要举措，并把首个千万千瓦级风电基地、光电基地布局在我市，这对推动酒泉经济跨越式发展提供了强力支撑；省委、省政府把我市作为"两翼齐飞"的重要"一翼"予以扶持，进一步增强了我们率先发展的信心；我市地域广阔，资源富集，区位条件较好，基础设施便利，是西部开发、承接东部产业西移、发展高载能产业的重要区域，只要我们抓住机遇，共克时艰，我们就一定能够发展得更快，发展得更好，提前实现全面建设小康社会的目标。

　　2010年政府工作的总体要求是：深入贯彻党的十七大和十七届三中、四中全会精神，以科学发展观为统揽，全面落实国家应对危机的一揽子计划，认真贯彻省委"中心带动、两翼齐飞、组团发展、整体推进"的区域发展战略，继续坚持"两抓整推"工作思路，着力打造"6＋2"产业集群，着力发展"一特四化"现代农业，着力统筹城乡协调发展，着力推进移民扶贫开发，着力保障和改善民生，推动全市经济平稳较快发展，促进社会和谐稳定。

　　2010年全市经济社会发展的预期目标是：地区生产总值增长12%，固定资产投资增长35%，财政收入增长12%，社会消费品零售总额增长20%，城镇居民人均可支配收入、农牧民人均纯收入增长10%以上，城镇登记失业率控制在4%以内，万元生产总值能耗降低5%，主要污染物排放减少2%，人口自然增长率控制在6.5‰以内。

　　2010年重点任务

　　为实现2010年工作目标，要着力抓好五个方面的工作：

　　一、以新能源建设为龙头，培育壮大优势产业

　　把新能源建设作为推动发展方式转变、提升区域经济实力的突破口，狠抓优势产业开发，提高经济运行质量，加快实现强市富民的目标。

　　1、在工业发展上，坚持以风电开发为牵引，全力实施振兴工业"6＋2"行动计划。打造"6＋2"产业集群，整体推进风电、光电、调峰电源、电网、装备制造、资源综合利用协调发展，抓好水能开发、核乏燃料后处理项目，实现工业发展新突破。新增风电装机300万千瓦，完成一期516万千瓦建设任务，做好二期755万千瓦风电项目招投标工作。建成敦煌20兆瓦、金塔23兆瓦光伏发电项目，酒泉热电厂首台机组投入运营，加快常乐电厂、红柳洼电厂、明沙窝电厂前期工作，新建、续建水电站12座，新增水电装机2万千瓦。着力解决电力输出瓶颈，建成750千伏输变电工程及配套电网，输电能力达到500万千瓦。提高风电产业配套水平，建设玉门"风光大道"，组建风电装备制造监测认证中心，争取天津鑫茂风机叶片、江苏九鼎机舱罩制造等项目建成投产，形成年产风力发电机组1000套、150万千瓦生产能力，装备制造业销售收入突破100亿元。依托新能源基地建设，大力发展循环经济和低碳经济，编制资源综合利用发展规划，推进金、钒等重点矿产资源整合，促进资源节约、集约利用。扶持中小企业发展，落实"五缓四降三补贴"扶持政策，加强银企合作，成立银企协会，完善信用体系，扩大信贷规模，新培育销售收入亿元以上工业企业5户，千万元以上工业企业20户，力争工业增加值增长18%以上。

　　2、在农业发展上，大力推进"一特四化"进程，构建酒泉现代农业新体系。认真落实"一特四化"专项规划，注重市场引导、政策扶持、中介服务、协调推进相结合，加快酒泉现代农业建设。优化种植结构，新增设施蔬菜1万亩，新建百亩以上特色生产基地100个，推广高效立体种植100万亩，新增万元田1万亩、5000元以上高效田3万亩、2000元以上高效田10万亩。突出农作物秸秆转化利用，青贮氨化率达到50%以上。继续抓好牛羊产业大县建设，提高草食畜牧业比重，新建10个种羊场，培育100个良种羊扩繁场，调引基础母羊2万只，新增肉羊50万只。做大做强农产品加工龙头企业，新增销售收入5000万元以上企业5个，农产品加工增值率达到45%。实施农业科技培训10万人（次），建设市级农业科技示范园区20个，建立农产品质量安全监管监测体系，推行农产品市场准入制度，农产品质量安全率达到98%以上。健全县乡村土地流转服务体系，促进和规范农村土地经营权流转，力争流转面积占到承包土地面积的10%。积极化解乡村债务，确保农民负担不反弹。

　　3、在三产发展上，坚持以线串点、以点带面，建设区域性商贸旅游中心。认真贯彻国家扩大消费、稳定出口的有关政策，发挥区位优势，加快商贸旅游业发展。一是积极争取项目资金，加快市场体系建设，扶持现代物流业、服务业和中小商贸企业发展，努力构建覆盖全市、辐射周边的流通网络。抓好春光市场、常青花木市场改造工程，力争时代广场、嘉怡电子市场、东关蔬菜市场建成运营。二是扩大农村消费市场，继续实施"万村千乡"和"双百市场"工程，做好家电、汽车和摩托车下乡工作，落实家电和汽车以旧换新政策，健全以城区配送中心为龙头、乡镇农家超市为骨干、村级农家店为基础的新型农村消费品流通网络。改革完善供销社流通服务体系，促进农产品快捷、安全、保质销售。三是加快旅游业发展。认真贯彻国务院关于加快发展旅游业的意见，编制旅游业振兴规划，制定扶持旅游业发展的政策措施，申报设立敦煌旅游经济特区，完善重点旅游景区建设规划，开工建设酒泉丝绸之路博览园二期工程、瓜州锁阳城和敦煌阳关文物景区二期工程，新建、创建一批高标准旅游饭店，鸣沙山·月牙泉景区创建为国家5A级景区。积极争取省上扶持政策，组建旅游汽车公司。推进旅游标准化建设，力争绿色旅游饭店达到50%以上。举办第五届敦煌飞天文化旅游节，拓展国内外旅游市场，力争接待海内外游客385万人次，增长10.5%，实现旅游收入27亿元，增长14.4%。

　　二、以项目建设为支撑，保持经济快速增长

　　认真研究国家应对危机的一揽子计划和推进西部开发的政策措施，及早编制"十二五"规划和相关专项规划，用规划统揽项目，用规划引领发展。充分发挥项目的支撑作用，以基础设施建设、生态环境保护、经济结构调整、社会事业发展、民生工程为重点，及时申报成熟项目，谋划前期项目，储备后续项目，争取更多的大项目、好项目列入国家和省上投资计划。在基础设施建设上，完善市域公路网络，加快瓜猩高速、敦当公路建设，力争"三北"通道酒泉段开工建设，新建酒泉、敦煌汽车客运枢纽站，做好酒嘉物流园区二期工程前期工作。实施市域铁路网中长期规划，争取敦格铁路、兰新铁路第二双线酒泉段开工建设，酒航铁路前期工作取得新的进展。拓展民航运输网络，完成敦煌机场改造，协调推进鼎新机场、下河清机场军民合用，争取将玉门、瓜州支线机场和肃北、阿克塞直升机备降点列入"十二五"民航机场建设规划。在生态建设上，启动敦煌水资源合理利用与生态保护综合规划，实施中小河流治理工程，完成鸳鸯、党河、洪临等4个灌区续建配套项目年度任务。扩大对外开放领域和招商引资规模，采取节会招商、以商招商、网上招商、跟踪招商等多种方式，大力引进高新产业项目，力争招商引资到位资金突破100亿元，增长18%。

　　三、以推进城镇化为依托，统筹城乡一体化发展

　　按照"建好城市、多予农村、流动畅通、普惠均等"的要求，促进城乡一体化发展。

　　1、坚持高起点规划。发挥规划在城乡建设中的导向作用，制定城乡规划管理办法，科学编制历史文化名城保护与建设规划，编制完成市、县土地利用总体规划和9个乡镇、68个村的村镇规划，加强城乡规划管理，用规划引领城乡统筹发展。

　　2、注重高标准建设。着力建设精品城市，提高城市综合承载能力，全面完成酒泉城区危旧住房拆迁改造，加快鼓楼东北片区建设，实施老城区防涝排洪工程，争取开工建设园林路、南环路、神舟路北段等道路工程，完成金泉路建设工程和滨河南路、世纪大道北段路灯架设。扩建第三水厂，解决新城区二期生产生活用水问题，理顺城市污水处理价格，酒泉污水处理改造升级工程建成运行。提高供热保障能力，加快实施酒泉热电联供和天然气入户工程，推进供热计量收费改革，抓好省、市试点，新建建筑必须安装供热计量装置，完成既有建筑供热计量改造26万平方米。实施中心城区增绿工程，新增公共绿地面积63.5万平方米，玉门、瓜州、肃北力争创建为省级园林城市。

　　3、实行精细化管理。探索城市管理市场化运行机制，加强城市道路、供热和物业管理，规范市政设施、园林绿化、环卫保洁标准。在社区、街道开展扁平化管理试点，发挥基层参与城市管理的能动性。深入开展市容环境综合整治活动，重点抓好城中村、城市出入口和城乡结合部的环境整治，净化城市及周边环境，积极创建全国文明城市。

　　4、推进一体化进程。在抓好城市建设的同时，编制全市城乡一体化发展规划。加大"三农"扶持力度，财政支农资金增长15%。认真落实惠农补贴政策，快捷办理支付手续。完善以奖代补措施，扶持葡萄、规模养殖、设施种植等高效产业发展。大力改善农村生产生活条件，狠抓村组道路硬化和居民住宅改造，推进"一池三改"沼气工程建设，继续开展村容村貌集中整治，新建社区化管理中心村20个，新增沼气用户2540户，建成村级服务网点57个。积极推进城乡公共服务和社会保障均等化，使农民在就学、就医、福利等方面得到更多的实惠。采取政府购买培训成果的方式，依托名企、名校培训劳务5万人，输转劳务14万人，形成政府、企业、学校相配合，培训、就业、创业相衔接的新格局。落实城乡同技同劳、同工同酬、同劳同保政策，力争户均输转1名劳动力，新增城市人口1万人，城镇化率提高1个百分点。

　　四、以节约用水为重点，加快生态文明建设

　　把节水作为生态建设的重中之重，促进生态修复，实现节能减排目标，提高资源保障水平和可持续发展能力。

　　1、推广高新节水技术。科学合理配置水资源，编制中长期水利建设规划，建设金塔、肃州地下水资源监测管理系统，推进水权、水价、用水制度市场化改革，逐步提高水价，加大节水投入，通过增效筹集节水资金，通过节水实现长远增效。继续实施百万亩节水灌溉工程，新增节水高效灌溉面积2万亩，新安装机井智能水表2000块，机井取水智能化控制率达到100%。

　　2、促进自然生态修复。按照"北拒风沙、南护水源、中建绿洲"的思路，加强自然生态保护，继续实施封滩育林、轮牧休牧工程，抓好祁连山水源涵养区保护及黑河流域治理。大力开展植树造林，力争完成人工造林10万亩，义务植树320万株。加大移民区农田林网建设和村镇绿化力度，改善移民区生态环境。推进集体林权制度改革，完成林业资源调查。

　　3、落实节能减排措施。实行环境影响评价和环保"三同时"制度，严把项目环评、能评、安评关，开展能效达标、节能评估和"双超双有"企业清洁生产强制审核。推广资源节约、替代和循环利用技术，开工建设大禹节水、银星纸业、禾麟粮油等一批节能、节水、资源综合利用项目，积极淘汰落后产能，万元GDP能耗下降5%，确保完成"十一五"节能20%的任务目标。着力抓好建筑节能和城市路灯节能工作，推行新建住宅太阳能集中供水，落实墙改措施，七县市城区全部禁止使用粘土实心砖。抓好工业园区、开发区及企业达标治理，重点源污染企业全部安装自动在线监控装置，化学需氧量和二氧化硫排放达到省控"十一五"目标。积极创建国家环保模范城市，开展城市大气、水、声及生态环境综合治理，加强疏勒河、党河、黑河流域生态监察，确保环境安全"零事故"。继续实施农村小康环保行动，制定"以奖代补"、"以奖促治"办法，开展农村集镇、道路、居民点环境卫生治理。编制完成30个乡镇的水源保护规划，划定农村饮用水源保护区，做好规模化养殖点污染防治工作。

　　五、以改善民生为根本，促进社会和谐稳定

　　加大资金投入，实施就业、教育、卫生、社会保障等惠民工程，着力改善民生。

　　1、千方百计扩大就业。充分发挥项目带动就业、创业促进就业、政府援助就业、培训提升就业的作用，用足用活就业政策，加大就业技能培训力度，购买公益性岗位1000个，对零就业家庭、下岗失业人员和"4050"人员实施重点援助和"一对一"帮扶计划，实现零就业家庭"动态清零"。采取政府补贴基本工资、企业发放岗位工资的形式，鼓励高校毕业生到企业就业。把自主创业作为就业的主渠道，小额贷款担保基金增加一倍，将贷款范围扩大到失业人员、复员转业军人、返乡农民工、城乡妇女等创业群体，新增城镇就业1万人以上，争取建成国家创业型城市。

　　2、全面发展社会事业。加快科技进步，建设创新型酒泉，继续实施科技特派员基层创业工程，开展"百团千人"服务企业科技行动，实施科技项目100项，示范推广实用新技术90项，申请专利120项。继续整合优化教育资源，全面完成教育布局初中阶段调整任务，做好寄宿制学校安全管理，建成市级标准化学校30所，酒泉第四、第五中学建成招生，新增办学规模7000人，应届初中毕业生高中阶段升学率保持在90%以上。提高公立医院财政补偿标准，妥善解决社区卫生服务中心业务经费和人员工资问题。开展创建"中医药工作示范县"活动，实施名院、名科、名医战略，启动敦煌市医院、瓜州县医院等标准化建设项目。完善突发公共卫生事件应急处置措施，高度重视甲型流感、鼠疫等重大疾病防控。推进市直文化系统体制改革，举办第九届广场文化艺术节，编制文化中心建设规划，新建5个移民乡镇综合文化站，"农家书屋"实现村级全覆盖。编制全市文物保护总体规划，做好丝绸之路"申遗"和第三次文物普查工作，实施西河滩遗址、晋城门、酒中古建筑群维护加固工程。深化人口与计划生育综合改革，统筹解决人口问题，稳定低生育水平。抓好体育竞技训练，组织参加全省第十二届运动会，承办全省第七届少数民族运动会和第八届残疾人运动会，完成体育场外围工程，开工建设市体育馆。启动手机电视、地面数字移动电视项目和玉门市、瓜州县数字电视整体转换工程，完成肃州、金塔、敦煌三县市"村村通"工程，广播电视覆盖率达到100%；提高数字电视转换率，酒泉城区数字电视用户达到4万户。实施"兴边富民"行动，深入开展民族团结进步宣传月活动，加大项目帮扶力度，促进民族团结、宗教和顺。提高统计数据质量和统计服务水平，开展第六次人口普查。建成马鬃山边防服务中心和防震减灾综合指挥中心，落实全省助残政策，做好双拥、市志、档案、外事、人防工作。

　　3、着力解决民生问题。全面推行城镇职工基本医疗保险、工伤保险、失业保险市级统筹，提高城镇居民医保和新农合筹资标准及报销比例，试行新农合门诊报销和城镇居民基本医疗保险门诊统筹办法，城乡居民住院费报销比例达到50%以上。推行城镇医疗保险刷卡结算，方便群众参保就医。全面实施被征地农民养老保险制度。坚持"阳光救助"，巩固城乡低保提标扩面成果，把符合条件的困难群众全部纳入保障范围。提高部分优抚对象补助标准，完善"三老一少"生活补贴和低保边缘户救助制度。加快农村敬老院、城市综合福利服务中心、救助管理站和流浪儿童保护中心建设。

　　切实解决民生问题，办好八件惠民实事：一是全面落实新医改政策，新农合人均筹资达到150元，恢复参合农民门诊报销政策，在基层医疗单位实行基本药物"零差价"；二是新增小额贷款担保基金2000万元，发放贷款1亿元，带动就业2万人；三是实施校舍安全工程，改造危旧校舍2万平方米，新建寄宿制学校公共浴室46座，实现寄宿制学校公共浴室全覆盖；四是继续实施农村安全饮水工程，解决5万名农村居民安全饮水问题；五是新建、改造农村道路600公里；六是新建廉租房1250套、经济适用住房1000套，发放租赁补贴1万户；七是改造农村危旧住房1.5万户、贫困残疾人住房300户，改建50个村级组织危旧办公用房；八是推行新型农村社会养老保险制度，金塔、阿克塞两个试点县参保率达到95%以上，确保符合参保条件的60岁以上老人应保尽保，人均发放月基础养老金55元。

　　4、加快移民扶贫开发。实施移民发展十年规划，强化项目支撑，争取资金不少于1亿元，重点抓好移民区土壤改良、道路林网和水利工程建设，改善移民生产生活条件。对现有土地进行测试评估，确保人均拥有1.5-2亩基本口粮田。因地制宜，发展种植、养殖、加工、流通等产业，按照每户培养一个科技明白人、每户输转一名劳动力的要求，分步实施劳动技能和种养技术培训，培训输转劳务2万人,力争移民人均纯收入增长20%以上。

　　5、促进社会安全稳定。深入开展安全生产执法行动，继续对矿山、危险化学品、道路交通、人员密集场所等重点领域实施专项整治，确保安全生产事故起数、死亡人数、受伤人数、经济损失四项指标控制在省控指标以内。加强信访工作，健全社会矛盾化解工作机制，治理违规违法上访。全面完成"五五"普法工作任务，强化法律服务和法律保障。深入开展公安"五大建设"和"三基"建设，抓好消防安全和道路交通安全管理，提高城市交通设施、视频监控、农村技防设备配套水平，完成公安、司法业务用房和基层"两所一庭"建设任务，确保实现"发案较少、秩序良好、社会稳定、群众满意"的目标。

　　各位代表，今年的工作任务十分艰巨，外部环境又复杂多变，能否完成全年的任务目标是对政府执政能力的重大考验。我们一定要扎实推进政府自身建设，加强与人大代表、政协委员和各界群众的沟通联系，充分吸纳群众智慧，提高行政决策水平。坚持依法行政，严格依法办事，规范政府议事规则和行政执法行为。认真履行党风廉政建设职责，打造公开、透明的"阳光"政府，注重从源头上预防和治理腐败，全面推行公务卡制度，严格执行招投标程序，深化财政审计、经济责任审计、政府投资项目审计和民生资金审计，坚决纠正损害国家和群众利益的不正之风，以良好的政风取信于民。

　　过去的一年，我们经受了严峻的考验，取得了辉煌的业绩；展望未来，我们面临着难得的机遇，肩负着神圣的使命。让我们在中共酒泉市委的坚强领导下，团结带领全市广大干部群众，攻坚克难，奋力拼搏，为全面完成"十一五"规划和市二届人大六次会议确定的任务目标而努力奋斗！
